# HSF1 Attenuates LPS-Induced Acute Lung Injury in Mice by Suppressing Macrophage Infiltration

**DOI:** 10.1155/2020/1936580

**Published:** 2020-12-18

**Authors:** Tao Li, Gui Xiao, Sipin Tan, Xueyan Shi, Leijing Yin, Chuyi Tan, Jia Gu, Yanjuan Liu, Huafei Deng, Ke Liu, Meidong Liu, Huali Zhang, Xianzhong Xiao

**Affiliations:** ^1^Key Laboratory of Sepsis Translational Medicine of Hunan, Department of Pathophysiology, Xiangya School of Medicine, Central South University, Changsha, Hunan 410008, China; ^2^Department of Pathophysiology, Medical College of Jiaying University, Meizhou, Guangdong 514031, China; ^3^Department of Nursing, Hainan Medical University, Haikou, Hainan 571199, China

## Abstract

Heat shock factor 1 (HSF1) is a transcription factor involved in the heat shock response and other biological processes. We have unveiled here an important role of HSF1 in acute lung injury (ALI). *HSF1* knockout mice were used as a model of lipopolysaccharide- (LPS-) induced ALI. Lung damage was aggravated, and macrophage infiltration increased significantly in the bronchoalveolar lavage fluid (BALF) and lung tissue of HSF^−/−^ mice compared with the damage observed in HSF1^+/+^ mice. Upon LPS stimulation, HSF^−/−^ mice showed higher levels of monocyte chemoattractant protein-1 (MCP-1) in the serum, BALF, and lung tissue and increased the expression of MCP-1 and chemokine (C-C motif) receptor 2 (CCR2) on the surface of macrophages compared with those in HSF1^+/+^. Electrophoretic mobility shift assays (EMSA) and dual luciferase reporter assays revealed that HSF1 could directly bind to heat shock elements (HSE) in the promoter regions of *MCP-1* and its receptor *CCR2*, thereby inhibiting the expression of both genes. We concluded that HSF1 attenuated LPS-induced ALI in mice by directly suppressing the transcription of MCP-1/CCR2, which in turn reduced macrophage infiltration.

## 1. Introduction

Acute lung injury is a type of acute and progressive hypoxic respiratory failure, where noncardiac pathogenic factors inside and outside the lungs cause damage to the alveolar and capillary endothelia, increasing the permeability of alveolar-capillary membrane. If the condition is not controlled, it will progress to acute respiratory distress syndrome (ARDS) (1) eventually leading to death. The pathogenesis of ALI is complex and has not been fully elucidated yet. Current studies indicate that inflammation is an important mechanism of ALI (2).

HSF1 is a transcription factor implicated in the heat shock response; it regulates the transcription of heat shock proteins such as Hsp27, Hsp60, and Hsp70, which play an important cytoprotective role in lung inflammation and injury (3–6). Our previous studies showed that HSF1 has a protective effect on LPS-induced multiorgan dysfunction syndrome (7, 8). Furthermore, HSF1 reduced leukocyte infiltration into the lungs and decreased the production of several inflammatory mediators, thereby attenuating inflammatory responses and exhibiting a protective effect on endotoxemia caused by LPS. However, the underlying mechanisms by which HSF1 alleviates ALI need to be further explored.

Macrophages are important effector cells in the pathogenesis of ALI. It has been reported that macrophage activation and migration are closely related to the severity of ALI (9). However, the effect of HSF1 on macrophages in ALI remains unclear. This study is aimed at exploring the effect of HSF1 on macrophages in ALI, by assessing the role of HSF1 in regulating chemokine expression. To the best of our knowledge, this is the first report showing that HSF1 alleviates LPS-induced macrophage infiltration and ALI in mice, by downregulating macrophage-related chemokines.

## 2. Materials and Methods

### 2.1. Animals

HSF1 knockout (HSF1^−/−^) and wild-type (HSF1^+/+^) mice were gifts from Dr. Ivor J. Benjamin (Medical College of Wisconsin, Milwaukee city, Wisconsin, USA) and have been described elsewhere (10). HSF1 mice were created using homologous recombination with a gene-targeting vector in embryonic stem cells as described by McMillan et al. (11). After mating HSF1 heterozygous female mice (♀HSF1^+/-^) with HSF1 heterozygous male mice (♂HSF1^+/-^), offspring with three genotypes was obtained: wild type, heterozygous type, and homozygous type. 3 weeks after birth, young mice were weaned and kept in separate cages. At the fourth week, mouse were genotyped using DNA from tail to identify qualified HSF1 knockout homozygous (HSF1^−/−^) and wild-type (HSF1^+/+^) mice for the experiments. Protocols for animal breeding and experiments were previously approved by the Institutional Animal Care and Use Committee of Central South University (Hunan, China) under license number 2018sydw0378 (approval date: 25 Nov., 2018).

### 2.2. Acute Lung Injury Model

Age- and sex-matched HSF1^−/−^ and HSF1^+/+^ mice (males, 4-5 months old, 20-25 g) were randomly divided into the HSF1^+/+^+normal saline (NS) group, HSF1^+/+^+LPS (E. coli 0111:B4, Sigma, USA) group, HSF1^−/−^ + NS group, and HSF1^−/−^ + LPS group. Mice were anesthetized with 5% chloral hydrate (0.01 ml/g, intraperitoneally) and 2% isoflurane (inhalation). Then, LPS (1 mg/ml) was instilled into the trachea at a dose of 3 mg/kg (12) in HSF1^+/+^+LPS and HSF1^−/−^ + LPS groups. HSF1^+/+^+NS and HSF1^−/−^ + NS groups were instilled with normal saline using the same dose and procedure as for LPS. Treatments were evaluated after 12 h, 24 h, and 36 h. In this experiment, pain and distress assessment of mice was conducted from postoperative to euthanasia. The method is as follows: mice were placed in a small transparent observation room (10 × 10 × 10 cm) for monitoring, and each mouse was observed for 3-4 minutes. According to GAP scores (13), pain indicators such as activity, posture, breathing pattern, coat condition, and relation to other mice were recorded, respectively. A blind observer assessed each mouse at 1, 6, 12, 24, and 36 h after the operation according to the time of sample collection. Mice were assigned a score of 0 (normal) or 1 (abnormal) for each parameter. Scores were recorded based on changes in pain indicators and then aggregated to get a composite score for each mouse. Mice in the LPS-treated group showed symptoms such as deeper breathing, decrease in movement, hair erect, back arch, reduced consumption of feed and water, and increased body temperature after using LPS. Therefore, in order to exclude the interference caused by LPS, we strictly observed differences between the LPS-treated group and the saline group and then scored the mice in the LPS-treated group.

### 2.3. Lung Wet/Dry Weight (W/D) Ratio

Pulmonary edema in treated groups was assessed using the lung wet/dry weight ratio. Mice were killed, and the lungs were removed and weighed (wet weight). Lungs were heated at 80°C for 24 h to obtain the dry weight and calculate the W/D ratio.

### 2.4. Extraction of Bronchoalveolar Lavage Fluid

BALF was collected as previously described (14). Mice were sacrificed by cervical dislocation. The skin of the neck was cut off to expose the trachea, and a small oblique incision was made at the telecentric end of the trachea using ophthalmic scissors. Then, a trocar was inserted into the trachea to wash mice lungs thrice by flushing 1 ml of cold sterile phosphate-buffered saline (PBS) per wash. The collected lavage fluid was centrifuged at 250× *g* for 15 minutes at 4°C. The cell pellet was resuspended in PBS for flow cytometry detection. The supernatant was stored at −80°C until further use.

### 2.5. Enzyme-Linked Immunosorbent Assay (ELISA)

Supernatants from lung tissue homogenate, BALF, and serum were collected to measure the levels of MCP-1 using an ELISA kit (MJE00B, RD, USA) according to the manufacturer's instructions.

### 2.6. Immunofluorescence

Immunofluorescence analysis was performed as previously published (15). Briefly, ALI model mice were sacrificed at different time points. The left lung was removed immediately after death and fixed in 4% paraformaldehyde for 24 h. After embedding in paraffin, the lung tissue was cut into 4 *μ*m thick sections. For antigen retrieval, sections were placed in sodium citrate buffer and heated in a microwave at 100°C for 15 min, then incubated with 10% bovine serum albumin (BSA) in PBS for 2 h at room temperature to block nonspecific binding sites. Subsequently, sections were incubated with murine macrophage-monocyte specific monoclonal antibody(anti-F4/80) (1 : 200, Santa Cruz, USA) and C-C chemokine receptor type 2 monoclonal antibody (anti-CCR2) (1 : 100, Abcam, USA) overnight at 4°C, followed by incubation with horseradish peroxidase secondary antibodies (1 : 50, ABclonal, Wuhan) for 1 h at 37°C under darkness. After washing 4 times with PBS, samples were treated with sealing liquid. Images were obtained using Panoramic 250/MIDI (Hungary).

### 2.7. Flow Cytometry

Cells collected from BALF and prepared for flow cytometric analysis as previously reported (16). Cells were suspended in 1 ml of 1× red blood cell lysis buffer (BD Biosciences, USA) for 5 minutes, then centrifuged at 250× *g* for 5 min. The supernatant containing BALF cells was resuspended in 100 *μ*l PBS, then blocked with 0.5 *μ*l of purified rat anti-mouse CD16/CD32 antibody (BD Biosciences, USA) for 10 min at 4°C. BALF cells were stained with fluorescent-conjugated antibodies: PerCP-Cy™ 5.5 rat anti-mouse CD45 antibody (0.5 *μ*l, BD Biosciences, USA), PE anti-mouse F4/80 antibody (0.5 *μ*l, BD Biosciences, USA), and Alexa Fluor 647 anti-mouse CCR2 (0.5 *μ*l, Biolegend, USA) for 20 min at 4°C under darkness, then centrifuged at 250× *g* for 5 min. The supernatant was fixed with 100 *μ*l of 2% paraformaldehyde at 4°C for 10 min under darkness. After washing twice with PBS, samples were resuspended in 200 *μ*l PBS, then analyzed by flow cytometry (BD Biosciences) using FlowJo analysis software (FlowJo, LLC, Ashland, Ore.).

### 2.8. Real-Time Quantitative Reverse Transcription PCR (qRT-PCR)

Total RNA extraction and qRT-PCR were performed as previously described (17).Total RNA was extracted from the lung tissue using TRIzol® reagent (Invitrogen, USA) according to the manufacturer's instructions, then retrotranscribed into cDNA using PrimeScript reverse transcriptase (TaKaRa, Kyoto, Japan) and random primers. qRT-PCR was performed using TB Green™ Premix ExTaq™ (TaKaRa, Kyoto, Japan) on a 7500 Real-Time PCR System (Applied Biosystems, USA). The primer sequences used for qRT-PCR were MCP-1 forward: 5′−TTA AAA ACC TGG ATC GGA ACC AA−3′ and reverse: 5′−GCA TTA GCT TCA GAT TTA CGG GT−3′; CCR-2 forward: 5′−ATC CAC GGC ATA CTA TCA ACA TC−3′ and reverse: 5′−CAA GGC TCA CCA TCA TCG TAG−3′; *β*-actin forward: 5′−TGC TGG AAG GTG GAC AGT GAG G −3′ and reverse: 5′−CAT TGC TGA CAG GAT GCA GAA GG−3′.The 2^-∆∆CT^ method was applied to determine relative mRNA expression levels.

### 2.9. Electrophoretic Mobility Shift Assay (EMSA)

EMSA was performed using the RAW 264.7 mouse macrophage cell line. Cells were treated at 43°C for 60 min in a 10 cm cell culture dish (11). After heating, nuclear extracts were prepared using nuclear and cytoplasmic protein extraction reagents (20126ES50, Yeasen, China). Oligonucleotide probes were synthesized and labeled with biotin by Sangon Biotech, which based on the sequence covering the HSE sequence (18, 19) from the promoter regions of *MCP-1* and *CCR2* (Table 1). The following oligonucleotide probes were used (for the sake of clarity, they are shown single-stranded, but double-stranded were used): MCP-1.1: 5′-aggtgagttttatata***GAA****A T****TTC***ttctgcaccatgagct-3′ (HSE1, −613 to −606 bp); MCP-1.2: 5′-atctcaggtccagggaagc a***TTC****TG****GAA***gcaccagcccca-3′ (HSE2, −1475 to −1468 bp); CCR2.1: 5′-agcatttaccta ***GAA****TT****TTC***cataacag-3′ (HSE1, −754 to −747 bp); CCR2.2: 5′-gtttacat***TTC****TA****GAA***cc ttatactgtg-3′ (HSE2: −1095 to −1088 bp). EMSA was performed using the LightShift® Chemiluminescent EMSA Kit (20148, Thermo Scientific, USA), according to the manufacturer's instructions. The binding reaction was performed as previously described (20). Briefly, biotin-labeled oligonucleotide containing the HSE sequence was incubated with 5 *μ*g of nuclear extract for 20 min at room temperature in binding buffer (10× binding buffer, 1 *μ*g Poly (dI-dC), 50% glycerol, 1% NP-40, 100 mM MgCl_2_, 100 mM EDTA). This reaction was then subjected to gel electrophoresis on a 5% native polyacrylamide gel and transferred to a nylon membrane. Biotin-labeled DNA was detected by chemiluminescence. For supershift assays, 2 *μ*l of anti-HSF1 antibody (ab2923, Abcam, USA) was added to the binding reaction. 200-fold molar excess of the unlabeled competitor oligonucleotides (competitive probe) and the unlabeled mutant oligonucleotides (mutant probe) were used for competition assays.

### 2.10. Dual-Luciferase Reporter Assay

The constitutively activated HSF1 plasmid pcDNA3.1 (+)/HSF1 (+) (mHSF1) and empty vector plasmid pcDNA3.1 (+)/HSF1-wt were kindly provided by Dr. Richard Voellmy at HSF Pharmaceuticals S.A., Switzerland. Generation of the *MCP-1* promoter luciferase reporter constructs (the full-length pGL3-MCP-1-wt (−1600 to −1 bp)), pGL3-MCP-1-Mut1 (mutations of HSE1, −613 to −606 bp), pGL3-MCP-1-Mut2 (mutations of HSE2, −1475 to −1468 bp), and pGL3-MCP-1-Mut3 (mutations of HSE1 and HSE2)), and the *CCR2* promoter luciferase reporter constructs (the full-length pGL3-CCR2-wt (−1162 to −1 bp), pGL3-CCR2-Mut1 (mutations of HSE1, −754 to −747 bp), pGL3-MCP-1-Mut2 (mutations of HSE2, −1095 to −1088 bp), and pGL3-MCP-1-Mut3 (mutations of HSE1 and HSE2), was performed by PCR and cloned into a luciferase vector pGL3-Basic. The authenticity of the synthesized sequence was verified by sequencing. Plasmids were extracted (D6950-01, Omega, USA) and stored at -20°C for subsequent experiments. RAW 264.7 cells were incubated in a 24-well plate and grown to 70-90% confluence, then transfected with 500 ng of the MCP-1 or CCR2 promoter luciferase reporter plasmid, 20 ng of pRL-TK, and 500 ng of mHSF1 plasmid or empty vector plasmid using the Lipofectamine™ 3000 transfection reagent (L3000015, Invitrogen™, USA). After 48 h of transfection, the cell lysate was extracted and tested using a dual-luciferase reporter assay kit (E1910, Promega, USA). Luminescence was measured using a Synergy^HI^ Microplate Reader (BioTek, USA).

### 2.11. Statistical Analysis

All data were analyzed using GraphPad Prism 7.0 (GraphPad Software, USA) and SPSS 21.0 (SPSS, USA). Data were expressed as mean ± standard deviation. Student's *t*-test was performed for comparing two groups and one-way analysis of variance (ANOVA) for comparing multiple groups, followed by a multiple comparison test (Bonferroni post hoc test). The Kaplan-Meier analysis was used to compare differences of survival rates between groups. *p* < 0.05 was considered statistically significant.

## 3. Results

### 3.1. HSF1 Alleviated Lung Tissue Damage and Improved Vascular Permeability in LPS-Induced ALI

**Figure 1:**
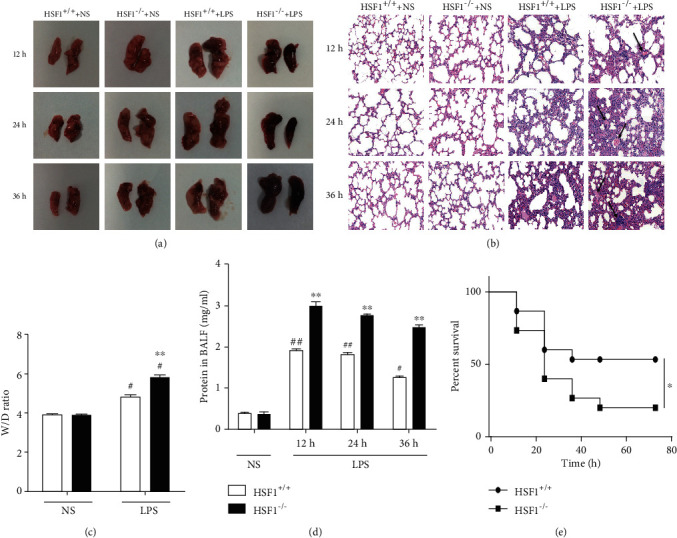
HSF1 reduced lung tissue injury and improved the outcome of LPS-induced ALI mice. (a) Effect of HSF1 on macroscopic changes at 12 h, 24 h, and 36 h after LPS treatment of ALI mice. (b) Representative morphological changes of the lung tissue from LPS-induced ALI mice. Arrows show pulmonary edema, inflammatory cell infiltration, and alveolar hemorrhage (HE staining, ×20 magnification). (c) The lung W/D ratio after 24 h of LPS treatment of ALI mice. (d) The total protein content in BALF from LPS-induced ALI mice; ^#^*p* < 0.05, ^##^*p* < 0.01, versus HSF1^+/+^+NS group; ^∗∗^*p* < 0.01, versus HSF1^+/+^+LPS group; *n* = 6 mice per group. (e) The survival rate of LPS-induced ALI mice expressed as a percentage. The survival rate was significantly lower in the HSF1^−/−^ group than in wild-type mice (20.0% versus 53.3%) after 72 h of LPS treatment; *n* = 15 mice per group; ^∗^*p* < 0.05, versus HSF1^+/+^ group. *p* values were determined using two-tailed Student's *t*-test for comparing two groups and one-way ANOVA for comparing multiple groups.

A dark red congestion was observed on the surface of the lungs from LPS-instillated mice, which was more obvious in the HSF1^−/−^ + LPS group than that in the HSF1^+/+^+LPS group (Figure 1(a)). Pathological histomorphology of the lungs revealed increased pulmonary edema, infiltration of inflammatory cells, alveolar hemorrhage, and destruction of the epithelial and endothelial cell structure in the HSF1^−/−^ + LPS group compared with the symptoms observed in the LPS-treated HSF1^+/+^ group (Figure 1(b)). The lung W/D ratio in the HSF1^−/−^ + LPS group was significantly higher (33%) than that in the HSF1^+/+^+LPS group (*p* < 0.01) (Figure 1(c)). The BALF total protein content at 12, 24, and 36 h after LPS instillation increases significantly in LPS-stimulated mice, showing a significant difference between HSF1^−/−^ + LPS and HSF1^+/+^+LPS groups (*p* < 0.01) (Figure 1(d)). Moreover, we found that the survival rate of the HSF1^−/−^ + LPS group was significantly lower than that of the HSF1^+/+^+LPS group (*p* < 0.05) (Figure 1(e)). These results showed that lung tissue injury was more severe in the HSF1^−/−^ group after LPS treatment, indicating that HSF1 alleviated lung tissue damage and improved vascular permeability and survival of LPS-induced ALI mice.

### 3.2. HSF1 Attenuated Macrophage Infiltration into BALF and Lung Tissue from LPS-Induced ALI Mice

**Figure 2:**
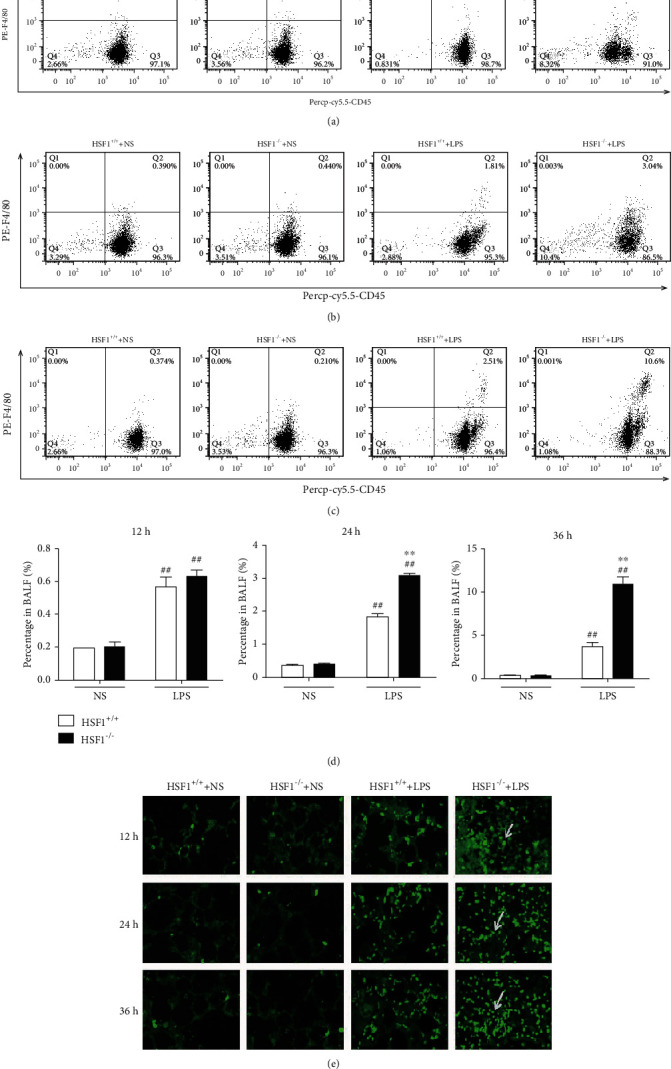
HSF1 reduced macrophage infiltration into the lung tissue from LPS-induced ALI mice. (a)–(d) BALF weas harvested and analyzed for macrophage accumulation using flow cytometry to detect CD45^+^ and F4/80^+^ cells at 12 h (a), 24 h (b), and 36 h (c) after LPS treatment. (d) Statistical analysis of (a), (b), (c), respectively. (e) Effect of HSF1 on macrophage infiltration into the lung tissue at 12 h, 24 h, and 36 h after LPS treatment in ALI mice (F4/80, green; white arrowheads indicate individual macrophages; immunofluorescence staining, ×40 magnification); ^#^*p* < 0.05, ^##^*p* < 0.01, versus HSF1^+/+^+NS group; ^∗^*p* < 0.05, ^∗∗^*p* < 0.01, versus HSF1^+/+^+LPS group; *n* = 6 mice per group. *p* values were determined using two-tailed Student's *t*-test for comparing two groups and one-way ANOVA for comparing multiple groups.

Macrophages are important effector cells in ALI pathogenesis and are closely related to disease severity. To evaluate the macrophage infiltration and exudation in the lung tissue from LPS-treated HSF1^+/+^ and HSF1^−/−^ mice, we measured the change in percentage of macrophages in BALF at different time points using flow cytometry. The results showed that at 12 h, 24 h, and 36 h after LPS instillation, the macrophage content in BALF from HSF1^−/−^ mice was significantly higher than that in HSF1^+/+^ mice (*p* < 0.01) (Figures 2(a)–(d)). Additionally, the tissue immunofluorescence assay results showed that the HSF1^−/−^ + LPS group had a significantly higher macrophage infiltration into the lung tissue than that observed in the HSF1^+/+^+LPS group (Figure 2(e)). These results suggested that HSF1 could attenuate macrophage infiltration into the lung tissue from LPS-induced ALI mice.

### 3.3. HSF1 Reduced the MCP-1 Expression in Serum, Lung Tissue, and BALF from LPS-Induced ALI Mice

**Figure 3:**
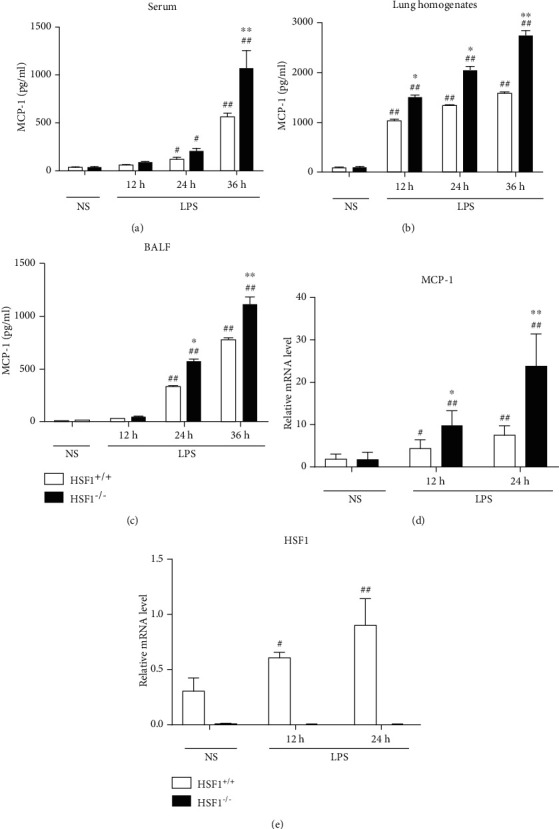
HSF1 attenuates the MCP-1 expression in serum, lung tissue, and BALF from LPS-induced ALI mice. MCP-1 levels were measured in (a) serum, (b) lung tissue, and (c) BALF at 12 h, 24 h, and 36 h after LPS treatment. (d) MCP-1 mRNA levels in the lung tissue from LPS-induced ALI mice using qRT-PCR. (e) HSF1 mRNA levels in the lung tissue from LPS-induced ALI mice using qRT-PCR. ^#^*p* < 0.05, ^##^*p* < 0.01, versus HSF1^+/+^+NS group; ^∗^*p* < 0.05, ^∗∗^*p* < 0.01, versus HSF1^+/+^+LPS group; *n* = 6 mice per group. *p* values were determined using two-tailed Student's *t*-test for comparing two groups and one-way ANOVA for comparing multiple groups.

Macrophage infiltration is regulated by MCP-1∕CCR2 chemokines (21–24). MCP-1 levels were measured at different time points in serum, lung tissue, and BALF. At 12 h, 24 h, and 36 h after LPS treatment, the MCP-1 levels in serum from the HSF1^−/−^ + LPS group peaked at 36 h and were significantly higher than those observed in the HSF1^+/+^+LPS group (*p* < 0.05), (*p* < 0.01) (Figure 3(a)). Similar changes were observed in the lung tissue and BALF (Figures 3(b) and 3(c)). Additionally, we found that MCP-1 mRNA levels in the lung tissue were significantly higher in the HSF1^−/−^ + LPS group than those found in the HSF1^+/+^ + LPS group (*p* < 0.05) (Figure 3(d)). Combined with the relative mRNA expression level of HSF1 (Figure 3(e)), these results indicated that HSF1 played a protective role in LPS-induced ALI mice by reducing the MCP-1 expression, thus inhibiting macrophage infiltration.

### 3.4. HSF1 Reduced the CCR2 Expression in Macrophages from LPS-Induced ALI Mice

**Figure 4:**
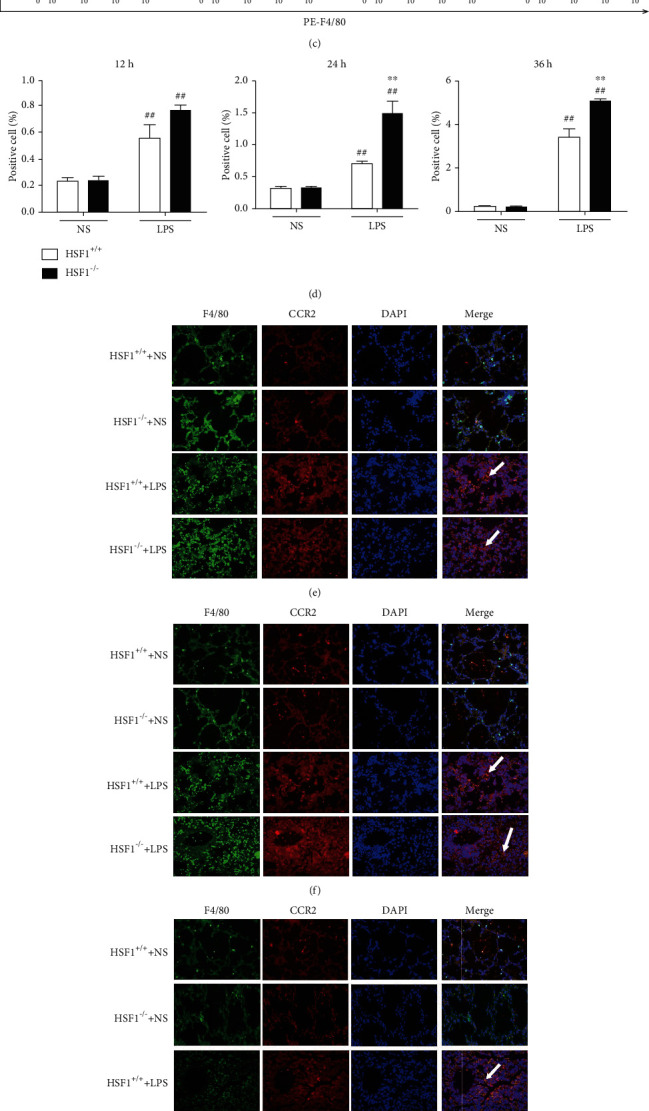
HSF1 reduced the CCR2 expression in macrophages from LPS-induced ALI mice. (a)–(d) BALF samples were analyzed using flow cytometry to measure the CCR2 expression in macrophages (CD45^+^, F4/80^+^, CCR2^+^) at 12 h (a), 24 h (b), and 36 h (c) after LPS treatment. (d) Statistical analysis of (a), (b), and (c). (e)–(g) Immunofluorescence staining of the CCR2 expression (red) in macrophages (green) from the lung tissue at 12 h (e), 24 h (f), and 36 h (g) after LPS treatment (F4/80, green; CCR2, red; DAPI, blue; yellow dots indicated by white arrowheads represent the CCR2 expression in macrophages; immunofluorescence staining, ×40 magnification). (h) CCR2 mRNA levels in the lung tissue from LPS-induced ALI mice using qRT-PCR. ^#^*p* < 0.05, ^##^*p* < 0.01, versus HSF1^+/+^+NS group; ^∗^*p* < 0.05, ^∗∗^*p* < 0.01, versus HSF1^+/+^+LPS group; *n* = 6 mice per group. *p* values were determined using two-tailed Student's *t*-test for comparing two groups and one-way ANOVA for comparing multiple groups.

Our next question was whether HSF1 affects the expression of CCR2 on the surface of macrophages. The CCR2 expression in mice BALF was detected using flow cytometry. We found that the CCR2 expression increased after 24 h and 36 h of LPS treatment, and the difference was more significant after 36 h (*p* < 0.01) (Figures 4(a)–(d)). Additionally, a tissue immunofluorescence assay was carried out to detect the expression of CCR2 in macrophages from the lung tissue. No significant difference was found between the expression levels of CCR2 in macrophages from HSF1^+/+^ and HSF1^−/−^ mice after 12 h of LPS treatment (Figure 4(e)). However, at 24 h and 36 h after LPS treatment (Figures 4(f) and 4(g)), the CCR2 expression in HSF1^−/−^ mice peaked and was higher than that in HSF1^+/+^ mice. Similarly, CCR2 mRNA levels in the lung tissue increased gradually after LPS stimulation, its value being significantly higher in HSF1^−/−^ mice than that in HSF1^+/+^ mice (*p* < 0.01) (Figure 4(h)). These results indicated that in the LPS-induced ALI model, HSF1 inhibited macrophage infiltration by reducing the expression of CCR2. On the other hand, we proved that by modifying the CCR2 expression, the macrophage infiltration increased significantly in the lung tissue from LPS-treated HSF1^−/−^ mice.

### 3.5. HSF1 Inhibited Monocyte/Macrophage Chemotaxis by Directly Regulating the Transcription of MCP-1/CCR2

**Figure 5:**
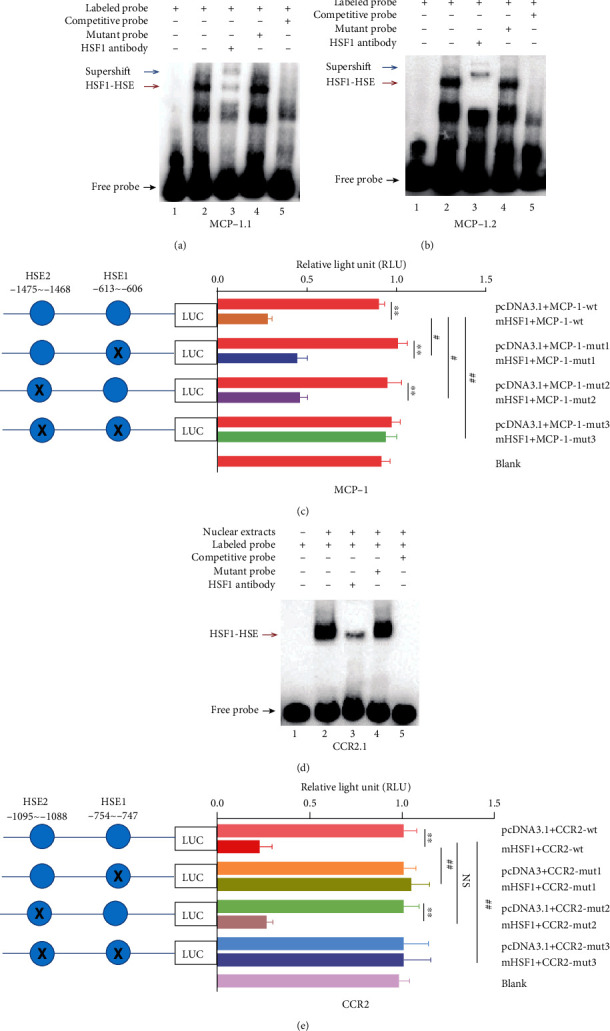
HSF1 downregulated the transcription of *MCP-1/CCR2*. (a, b, d) The binding of HSF1 to HSE in *MCP-1* and *CCR2* promoters was assessed using EMSA. (a, b) EMSA was used to detect the binding of the HSF1 protein with HSE1 (a) and HSE2 (b) in the *MCP-1* promoter region in vitro. The biotin probe specific for HSE1 or HSE2 was bound by nuclear extracts of RAW 264.7 cells, which could be blocked by an HSF1 antibody. (d) EMSA was used to detect the binding of the HSF1 protein with HSE1 in the *CCR2* promoter region in vitro. The biotin probe specific for HSE1 was bound by nuclear extracts, which could be blocked by an HSF1 antibody. Lane 1: negative control; lane 2: biotin-labeled HSE probe preincubated with nucleoproteins (HSF1-HSE complexes); lane 3: supershift analysis using anti-HSF1 antibodies; lane 4: competition using a 200-fold excess of the unlabeled competitive probe; lane 5: competition using a 200-fold excess of the unlabeled mutant probe. The HSF1/HSE complex is indicated by red arrowheads, the supershift band is indicated by blue arrowheads, and unbound HSE probe is indicated by black arrowheads. (c, e) Luciferase reporter assay analysis of HSF1 regulation on the promoter transcription activity of *MCP-1* (c) and *CCR2* (e) in RAW 264.7 cells transfected with the HSF1 overexpression vector (mHSF1) or empty expression vector pcDNA3.1. After 48 h of transfection, the cell lysate was extracted, and the firefly luciferase activity was measured and normalized to the Renilla luciferase activity. ^∗∗^*p* < 0.01, pcDNA3.1 versus mHSF1 (intragroup comparison); ^#^*p* < 0.05, versus mHSF1 + MCP-1-wt (or mHSF1 + CCR2-wt); ^##^*p* < 0.01, versus mHSF1 + MCP-1-wt (or mHSF1 + CCR2-wt). Data are representative of at least three independent experiments. *p* values were determined using two-tailed Student's *t*-test for comparing two groups and one-way ANOVA for comparing multiple groups.

In previous studies, we prepared an endotoxemia model using HSF1^−/−^ and HSF1^+/+^ mice. We prepared a microarray containing 384 inflammatory factor genes for screening HSF1-regulated inflammation-associated genes. It was found that HSF1 inhibited the expression of MCP-1 and its receptor CCR2 (25). A further bioinformatic analysis identified two HSE (HSE1 and HSE2) in the promoters of *MCP-1* and *CCR2*. To conduct the EMSA, we designed probes recognizing the identified HSE sites. Our results showed that HSF1 specifically bound in vitro to HSE1 in both promoters and to HSE2 in the promoter of *MCP-1* (Figures 5(a), (b), and (d)). To investigate whether HSF1 regulates the *MCP-1* and *CCR2* expression at the transcriptional level, we performed luciferase assays using reporter constructs containing a DNA fragment with HSE (wt) or with mutant HSE of the *MCP-1* and *CCR2* promoters, both separately and together (Figures 5(c) and 5(e)). As shown in Figure 5(c), the HSF1 overexpression resulted in significantly inhibited the promoter activity of MCP-1-wt compared with that observed for the empty vector (*p* < 0.01). The promoter activity of *MCP-1* was still suppressed by the single HSE1 or HSE2 mutant reporter structure (*p* < 0.05). However, HSF1 did not alter the promoter activity when both two sites were mutated simultaneously. Combined with EMSA results, these findings suggested that the HSF1-binding sites at −613 to −606 bp (HSE1) and−1475 to −1468 bp (HSE2) played a major role in regulating the *MCP-1* promoter activity by HSF1. On the other hand, the luciferase activity of the CCR2-wt reporter was repressed in cells cotransfected with mHSF1 plasmid compared with that observed in the empty vector (Figure 5(e)). However, the repression could be removed when HSE1 was mutated, but not the mutations of HSE2. Combined with EMSA results, these findings suggest that the HSE1 (−754 to −747 bp) played a major role in regulating the *CCR2* promoter activity by HSF1. Overall, these results indicated that HSF1 could downregulate the transcription of *MCP-1* and *CCR2*, by binding to HSE in their promoter regions.

## 4. Discussion

ALI, or its more severe phenotype acute respiratory distress syndrome (ARDS), can be caused by factors such as severe trauma or infection (26). ARDS affects approximately 200,000 patients every year, resulting in nearly 75,000 deaths in the USA (27). The pathogenesis of ALI is complex and has not been fully elucidated yet. Therefore, further understanding of its pathogenesis has potential therapeutic significance. Based on an ALI model induced by intratracheal instillation of LPS in HSF1 knockout mice, this study showed that HSF1 had a protective effect on ALI mice. To the best of our knowledge, we demonstrated for the first time that the protective effect of HSF1 is due to the downregulation of *MCP-1* and *CCR2*, owing to the binding of HSF1 to HSE in the promoters of both genes. Consequently, macrophage infiltration was suppressed.

Our previous work showed that HSF1 alleviated multiple organ damages by inhibiting the release of inflammatory factors and leukocyte infiltration into the tissue of endotoxemia mice (7, 8). To determine the protective role of HSF1 in LPS-induced ALI, we used HSF1 knockout mice to prepare an ALI model. We found that after LPS stimulation, the lung tissue damage in HSF-1^−/−^ mice was significantly more severe than that observed in wild-type mice. Furthermore, HSF1 alleviated pulmonary edema, macrophage infiltration, alveolar hemorrhage, reduced protein leakage, and improved the survival of ALI mice. These observations are consistent with those from other studies using LPS-induced ALI models (28, 29). Altogether, these results demonstrated that HSF1 could alleviate lung damage, improved the survival rate, and had a protective effect against LPS-induced ALI.

The inflammatory response is a crucial element in the pathogeneses of ALI; its pathophysiological features are inflammatory exudation and an imbalance between proinflammatory and anti-inflammatory responses, which may eventually lead to respiratory failure (30). The evidence suggests that macrophages are key cells in the pathogenesis of ALI/ARDS (31, 32). Macrophage recruitment into the damaged tissue is an important process leading to inflammatory damage. Some studies show that MCP-1 and its receptor CCR2 play important roles in macrophage migration (21–24). MCP-1 (also known as CCL2) is a member of the C-C chemokine family and binds to CCR2 (33). MCP-1 is usually secreted by macrophages in response to pathogen infection (34) and is the most potent inducer of the signal transduction pathways leading to monocyte transmigration (35). The high affinity of MCP-1 for CCR2 is an important factor in promoting monocyte/macrophage activation, chemotaxis, and inflammatory responses, resulting in important biological consequences (21, 22). To confirm the effect of HSF1 on macrophage infiltration, we analyzed serum, lung tissue homogenates, and BALF from ALI mice to quantify the concentration of MCP-1. Further, we analyzed the expression of CCR2 on the surface of macrophages in BALF and lung tissue using flow cytometry and immunofluorescence assay, respectively. The results showed that the expression of MCP-1 in serum, lung tissue, and BALF from LPS-stimulated HSF1^−/−^ mice gradually increased after stimulation and was significantly higher than that observed in wild-type mice. The expression of CCR2 on the surface of macrophages was concomitant with the MCP-1 expression.

The results suggested that HSF1 inhibited macrophage migration by downregulating both MCP-1 and CCR2. However, the downregulating mechanism is unclear. It has been proved that HSF1 plays a role in several diseases by regulating gene transcription (36–38). The binding of HSF1 to a gene promoter is a key step to activate gene transcription. Under stress conditions, HSF1 forms homotrimers that upon phosphorylation bind to the promoter of heat shock genes to regulate their transcription (39–41). HSF1 can regulate the expression of other genes, including inflammatory cytokines (37, 42, 43). To explore the effect of HSF1 on inflammation, in previous studies, we screened for inflammation-associated genes potentially regulated by HSF1, using a microarray containing inflammatory cytokine genes. We found that HSF1 regulated the expression of several genes including *MCP-1/CCR2* (25). Further, bioinformatic analysis showed that there were several HSE in the promoters of *MCP-1/CCR2*. Combined with the results from the JASPAR core database, we proposed a hypothesis on how HSF1 may affect the course of inflammation by directly regulating the expression of *MCP-1/CCR2*. In this study, we tested such hypothesis by using EMSA and dual luciferase reporter assay experiments and found that HSF1 could directly downregulate *MCP-1/CCR2*. Therefore, the protective effect of HSF1 on ALI mice would be explained by the inhibition of macrophage migration and infiltration, owing to the downregulation of *MCP-1/CCR2*.

## 5. Conclusion

In summary, HSF1 attenuated LPS-induced ALI in mice by suppressing macrophage infiltration owing to the downregulation of *MCP-1/CCR2*.

## Figures and Tables

**Table 1:** List of MCP-1 and CCR2 probe sequences.

Probe	Probe sequence(5′-3′)
MCP-1.1:labeled probe F:	Biotin-AGGTGAGTTTTATATAGAAATTTCTTCTGCACCATGAGCT
MCP-1.1: labeled probe R:	Biotin-AGCTCATGGTGCAGAAGAAATTTCTATATAAAACTCACCT
MCP-1.1: competitive probe F:	AGGTGAGTTTTATATAGAAATTTCTTCTGCACCATGAGCT
MCP-1.1:competitive probe R:	AGCTCATGGTGCAGAAGAAATTTCTATATAAAACTCACCT
MCP-1.1: mutant probe F:	ATGTGAGTGCTATCTAGCACTGTATGCTGCACAATGAGGT
MCP-1.1: mutant probe R:	ACCTCATTGTGCAGCATACAGTGCTAGATAGCACTCACAT
MCP-1.2: labeled probe F:	Biotin-ATCTCAGGTCCAGGGAAGCATTCTGGAAGCACCAGCCCCA
MCP-1.2: labeled probe R:	Biotin-TGGGGCTGGTGCTTCCAGAATGCTTCCCTGGACCTGAGAT
MCP-1.2: competitive probe F:	ATCTCAGGTCCAGGGAAGCATTCTGGAAGCACCAGCCCCA
MCP-1.2:competitive probe R:	TGGGGCTGGTGCTTCCAGAATGCTTCCCTGGACCTGAGAT
MCP-1.2: mutant probe F:	ATATCAGATCCATGGCAGCAGTCTGTACGTACTAGACTCA
MCP-1.2: mutant probe R:	TGAGTCTAGTACGTACAGACTGCTGCCATGGATCTGATAT
CCR2.1:labeled probe F:	Biotin-AGCATTTACCTAGAATTTTCCATAACAG
CCR2.1: labeled probe R:	Biotin-CTGTTATGGAAAATTCTAGGTAAATGCT
CCR2.1:competitive probe F:	AGCATTTACCTAGAATTTTCCATAACAG
CCR2.1:competitive probe R:	CTGTTATGGAAAATTCTAGGTAAATGCT
CCR2.1: mutant probe F:	AGCACTGATCTATGATTAATCATGACAG
CCR2.1: mutant probe R:	CTGTCATGATTAATCATAGATCAGTGCT
CCR2.2: labeled probe F:	Biotin-GTTTACATTTCTAGAACCTTATACTGTG
CCR2.2: labeled probe R:	Biotin-CACAGTATAAGGTTCTAGAAATGTAAAC
CCR2.2: competitive probe F:	GTTTACATTTCTAGAACCTTATACTGTG
CCR2.2:competitive probe R:	CACAGTATAAGGTTCTAGAAATGTAAAC
CCR2.2:mutant probe F:	GTCTATAGGTCTATCACTCTATGCTGTG
CCR2.2:mutant probe R:	CACAGCATAGAGTGATAGACCTATAGAC

## Data Availability

The data used to support the findings of this study are available from the corresponding author upon request.

## Abbreviations

ALI: Acute lung injury
ARDS: Acute respiratory distress syndrome
BALF: Bronchoalveolar lavage fluids
BSA: Bovine serum albumin
CCR2: Chemokine (C-C motif) receptor 2
ELISA: Enzyme-linked immunosorbent assay
EMSA: Electrophoretic mobility shift assay
HSE: Heat shock element
HSF1: Heat shock factor 1
LPS: Lipopolysaccharide
MODS: Multiorgan dysfunction syndrome
MCP-1: Monocyte chemoattractant protein-1
NS: Normal saline
PBS: Phosphate-buffered saline
qRT-PCR: Real-time quantitative reverse transcription PCR
W/D: Wet/dry weight.
